# Out of Their Depth? Isolated Deep Populations of the Cosmopolitan Coral *Desmophyllum dianthus* May Be Highly Vulnerable to Environmental Change

**DOI:** 10.1371/journal.pone.0019004

**Published:** 2011-05-18

**Authors:** Karen J. Miller, Ashley A. Rowden, Alan Williams, Vreni Häussermann

**Affiliations:** 1 Institute of Marine and Antarctic Studies, University of Tasmania, Hobart, Tasmania, Australia; 2 National Institute of Water and Atmospheric Research, Wellington, New Zealand; 3 Commonwealth Scientific and Industrial Research Organisation, Marine and Atmospheric Research, Hobart, Tasmania, Australia; 4 Facultad de Recursos Naturales, Escuela de Ciencias del Mar, Pontificia Universidad Católica de Valparaíso, Valparaíso, Chile; 5 Huinay Scientific Field Station, Chile; National Oceanic and Atmospheric Administration/National Marine Fisheries Service/Southwest Fisheries Science Center, United States of America

## Abstract

Deep sea scleractinian corals will be particularly vulnerable to the effects of climate change, facing loss of up to 70% of their habitat as the Aragonite Saturation Horizon (below which corals are unable to form calcium carbonate skeletons) rises. Persistence of deep sea scleractinian corals will therefore rely on the ability of larvae to disperse to, and colonise, suitable shallow-water habitat. We used DNA sequence data of the internal transcribed spacer (ITS), the mitochondrial ribosomal subunit (16S) and mitochondrial control region (MtC) to determine levels of gene flow both within and among populations of the deep sea coral *Desmophyllum dianthus* in SE Australia, New Zealand and Chile to assess the ability of corals to disperse into different regions and habitats. We found significant genetic subdivision among the three widely separated geographic regions consistent with isolation and limited contemporary gene flow. Furthermore, corals from different depth strata (shallow <600 m, mid 1000–1500 m, deep >1500 m) even on the same or nearby seamounts were strongly differentiated, indicating limited vertical larval dispersal. Genetic differentiation with depth is consistent with the stratification of the Subantarctic Mode Water, Antarctic Intermediate Water, the Circumpolar Deep and North Pacific Deep Waters in the Southern Ocean, and we propose that coral larvae will be retained within, and rarely migrate among, these water masses. The apparent absence of vertical larval dispersal suggests deep populations of *D. dianthus* are unlikely to colonise shallow water as the aragonite saturation horizon rises and deep waters become uninhabitable. Similarly, assumptions that deep populations will act as refuges for shallow populations that are impacted by activities such as fishing or mining are also unlikely to hold true. Clearly future environmental management strategies must consider both regional and depth-related isolation of deep-sea coral populations.

## Introduction

The impact of climate change on marine ecosystems is likely to be large [1]. Scleractinian or stony corals are considered to be particularly vulnerable to climate mediated environmental changes due to the effects of ocean acidification on calcifying marine organisms (see special issue introduced by [2]). Stony corals require calcium carbonate in the form of aragonite to build their skeletons, which they obtain from seawater where carbonate is in solution. As the ocean acidifies, carbonate concentration in seawater may decline to a point where seawater is no longer saturated with this essential mineral [3], with potentially deleterious results for corals.

Deep-sea or cold-water stony corals are considered to be at greatest risk from ocean acidification because deep ocean regions (i.e. 1000 s of metres depth) will rapidly become uninhabitable as the Aragonite Saturation Horizon (ASH) (the interface between over- and under-saturation of aragonite) rises. Climate change models predict that the ASH will become shallower as the oceans acidify and temperatures increase. By 2099 the ASH is predicted to be situated at <400 m depth throughout most of the Southern Ocean [4] and 70% of the locations where cold water corals occur globally are predicted to be affected by 2100 [5].

The ability of deep-sea stony corals to persist in the face of climate change will depend both on their ability to survive in shallower water, but also their ability to disperse into shallow habitats as the ASH rises. The shallower summits of seamounts have been predicted to offer deep-sea corals a proximal refuge from the effects of ocean acidification [6]. However, recent genetic data suggest depth may be an important isolating mechanism for deep-sea populations on seamounts within a region [7], including for some deep-sea corals [8], [9]. If this is true, and there are limited connections between deep and shallow populations of a species even on seamounts in the same geographic region, then deep populations and the unique genetic diversity they contain may well be lost as ocean climate changes.

The scleractinian coral *Desmophyllum dianthus* is a widespread deep-sea coral species, with a cosmopolitan distribution from the sub-Antarctic to the North Sea [10]. Although strictly a solitary coral, *D. dianthus* can form important reef-like structures in areas where it is abundant (e.g. [11]) and thus plays a central role as an ecosystem engineer by providing habitat for other organisms. Importantly, *D. dianthus* has a large depth range extending to 2500 m depth on seamounts and the continental slope, but with emergent populations as shallow as 4 m in the fiords of New Zealand and Chile [12], [13]. It thus represents an excellent species to test for evidence of isolation in deep sea ecosystems. Here we use a combination of DNA sequence data and morphological data for *Desmophyllum dianthus* to 1) explore the relationship between geographically isolated populations in the Southern Hemisphere and 2) determine the role of depth as a mechanism for isolating populations.

## Methods

### Samples, species and study area

**Figure 1:**
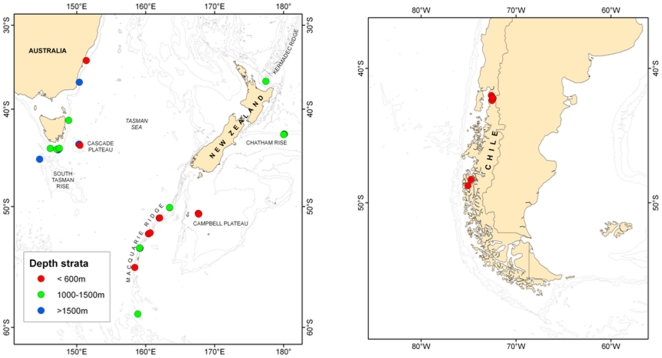
Maps showing the location of *Desmophyllum dianthus* sample sites (a) off SE Australia and New Zealand, and (b) in the fiords of Chile. Note: Symbols for some sites are overlapping, including sites in different depth strata on the same seamount feature.

We examined samples of *D. dianthus* from three geographic regions in the southern hemisphere: SE Australia, New Zealand and Chile (Fig. 1). Australian material incorporated sites from off the coast of New South Wales to Tasmania and included specimens from the Australian Museum as well as samples collected recently as part of two scientific voyages off southern Tasmania (SS200702 and TN288 which deployed the Jason ROV). New Zealand samples were from the Kermadec Ridge in the north to the Macquarie Ridge in the south and included specimens from the NIWA Invertebrate Collection and material collected as part of two recent scientific voyages (TAN0604 and TAN0803). Chilean material was collected using SCUBA in 2006 from the deep-water emergent populations in the Patagonian fiords, and range from Fjord Comau in the north (approx 42°S) to Seno Waldemar (approx. 48°S) in the south. Corals from recent collections were preserved in either >90% ethanol or at −80°C for genetic analysis.

The total of 162 individual specimens used in the study were from 18, 21 and 9 sites in the SE Australia, New Zealand and Chile regions, respectively. Samples were from a range of depths, which we grouped into three depth strata based on preliminary data exploration: shallow (<600 m), mid (1000–1500 m) and deep (>1500 m). Samples were from all three depth strata for SE Australia, all but the deep stratum for New Zealand, and only the shallow stratum for the Chilean region. In the SE Australian and New Zealand regions, most samples were from seamounts, some of which were from the same or physically close seamounts but from different depth strata (i.e. Cascade Plateau, Fig. 1, Table S1).

### Molecular protocols

Genomic DNA was extracted from coral tissue with the Qiagen DNeasy Kit according to the manufacturer's specifications, but with an extended period of lysis (overnight incubation at 55°C). We targeted three different DNA regions, incorporating nuclear and mitochondrial markers with varying rates of mutation; the mitochondrial ribosomal subunit (16S) using the scleractinian-specific primers LP-16S-F and LP-16S-R [14], the Internal Transcribed Spacer region (ITS, spanning the ITS1, 5.8S and ITS2) using the universal primers ITS4 and ITS5 [15], and the Mitochondrial Control Region (MtC) using a mix of the scleractinian-specific primers Mt-Coral-Fwd-1, Mt-Coral-Fwd-2, Mt-Coral-Rev-1, Mt-Coral-Rev-2 [8].

PCR reactions (50 µl) contained DNA template, 1× thermophilic DNA Polymerase buffer (Bioline), 1.5 mM MgCl2 (Promega), 3 U RedTaq DNA polymerase (Bioline), 0.2 µM of each primer (Sigma-Proligo), 80 µM of each dNTP and 0.5 µg Bovine Serum Albumen (Promega). The thermal cycling profile consisted of an initial denaturation at 95°C for 5 min, then 35 cycles of a three-step program (95°C for 30 sec, 55°C for 30 sec and 72°C for 1 min), with a 10 min final extension at 72°C. PCR products were purified using the QIAquick PCR Purification kit (Qiagen) prior to sequencing on an ABI3730XL automated sequencer.

Sequences were analysed either with Sequencher 4.5 software or MEGA3.1 [16], and consensus sequences generated for each sample using forward (5′-3′) and reverse (3′-5′) primer sequences. BLAST searches were performed for a subset of sequences to ensure that the correct gene region had been amplified and that sequences closely matched data from other coral groups. Sequences for each DNA region were aligned using the ClustalW alignment algorithm implemented in MEGA3.1 [16], and reduced to a consistent length across all individuals. The lengths of sequences used in subsequent analyses were: 16S - 308 bp, ITS – 581 bp, MtC – 258 bp. All sequences generated in this study were lodged with GenBank (Accession Nos HM015301–HM015310, HM015339–HM015347, and JF827609–JF827643).

### Sequence Data analysis

For data analysis, samples were pooled into “populations” representing all corals from sites within a region/depth to ensure adequate replication for statistical comparison and also because we have shown previously that DNA sequence does not resolve genetic structure among sites within geographic regions for deep sea corals [8]. We calculated genetic diversity measures for each of the populations as well as across the whole data set using Arlequin 3.01 [17], along with tests for selective neutrality (calculated as Tajima's *D* and and Fu's *F_s_*) with significance levels based on 10,000 permutations.

To explore the genetic relationships among *D. dianthus* populations we created parsimony networks in TCS 1.21 [18] for each of the three DNA regions and using 95% connection limit between haplotypes. Geographic data was overlayed on the resulting network to determine groupings within the data. We subsequently tested for evidence of genetic subdivision among geographic regions and depth strata by Analysis of Molecular Variance (AMOVA) using the software package Arlequin 3.01 ([17]). Pairwise values of *F_ST_* were also calculated in Arlequin 3.01 using Exact tests and based on a Markov Chain length of 10,000. We used subsequent values of *F_ST_* to estimate gene flow among populations as *N_e_m* = (1/*F_ST_*−1)/4 [19].

Estimates of gene flow based on *F_ST_* are limited by the underlying assumption of an island model whereby all populations exchange migrants; an assumption that is rarely met in natural populations. We therefore used LAMARC V2.1.5 [20] to generate coalescent estimates of migration rates among all populations using a Bayesian framework. Because it is not possible to combine data that have recombination with data that do not in a single analysis, we analysed nuclear ITS in one analysis and the two mitochondrial regions (16S and MtC) in a second analysis but allowing mutation rates to vary. We used jModelTest [21] to determine the most appropriate substitution model for each DNA region and these were used to guide model choice in LAMARC. Additionally, transition/transversion (TT) ratios for each DNA region were calculated in PAUP V4.0 [22]. The final models used were; ITS - Jukes Cantor (run in LAMARC using F84 model with all nucleotide frequencies set to 0.25 and TT ratio set to 0.500001) and incorporating recombination; MtC - HKY+G (used F84 model with TT ratio of 2.9) and 16S - F81 (used F84 and TT ratio set to 0.500001). Exploratory analyses in LAMARC were run with default starting values for Theta (Θ), Migration (*M*) and recombination (*r*) and with uniform prior distributions. The exploratory runs consisted of 5 initial chains, 1 final chain, 100,000 steps and a burn-in of 10,000 steps. Additionally we used two simultaneous searches with adaptive heating and temperatures set to 1 and 1.5. The estimates of Θ and *M* (for all three DNA regions) and *r* (for ITS only) from three preliminary runs were averaged and used to set the start values for the final analysis which consisted of one long chain, 10,000,000 steps, a 10,000 step burn-in, and 3 replicates. In addition we plotted the curve files from each final run to confirm that sufficient steps had been performed to ensure a single optimum was estimated for each parameter. We also calculated *N_e_m* from the LAMARC estimates of migration for comparison with *F_ST_*-based estimates by multiplying effective population size (Θ) by migration (*M*).

### Morphological analysis

*Desmophyllum dianthus* supposedly has a cosmopolitan distribution, although there have been at least 11 species described that are now synonymised to this single species [23]. One of the greatest challenges in the delineation of *Desmophyllum* species is the relative paucity of taxonomic features. Where coral populations are potentially isolated, the possibility that allopatric speciation may occur is high, and so it may be that isolated populations of *Desmophyllum* have diverged or be in the process of speciation. To determine if there was morphological divergence in line with geographic isolation and genetic differentiation, we used a quantitative morphometric approach to compare skeletal characteristics of *D. dianthus* from a subset of specimens from the three geographic regions. We measured nine skeletal characters in 86 randomly chosen *D. dianthus* individuals (18, 27 and 41 from SE Australia, New Zealand and Chile respectively). Characters measured included: corallite height, corallite length, corallite width, total number of septa, number of septal cycles, septa height (mean of 5 measurements/corallite), septa width (mean of 5 measurements/corallite), septa thickness (mean of 5 measurements/corallite), number of costal cycles and costae length (mean of 5 measurements/corallite). Because *D. dianthus* most likely displays ontogenetic change in skeletal morphology (i.e. polyps grow taller and larger through time) we standardised measurements for each coral to account for possible age variation in corals collected within each region; coral “size” was determined as the ratio of corallite area to corallite height (where area was calculated as π × (length × width)/2), total number of septa was standardised to corallite area (septa/area) and septa height, width and thickness were standardised by corallite size (height/size; width/size, thickness/size) and costae length was standardised by corallite height (costae length/corallite height). Final analysis was based on these 6 standardised parameters along with the 2 raw data parameters - number of septal cycles and number of costal cycles. We tested for variation in skeletal characters of *D. dianthus* individuals among the three geographic regions using Multivariate Analysis of Variance (MANOVA), and we used Canonical Discriminant Analysis (CDA) to visually examine any morphological differences in *D. dianthus* from the three regions.

## Results

### Genetic diversity

16S was the least variable of the three DNA regions sequenced. The final 16S data set included 308 bp of sequence from 90 individuals, but contained only 11 haplotypes. There were only 9 variable sites (2.9% variation) with the mean number of pairwise differences between sequences 1.08±0.72. Overall nucleotide diversity was also low (π = 0.004±0.003), but varied considerably across the three geographic regions (Table 1).

**Table 1:** Genetic diversity measures for *Desmophyllum dianthus* from SE Australia, New Zealand and Chile collected from shallow (<600 m), mid (1000–1500 m) and deep (>1500 m) depth strata.

	16S				MtC				ITS			
	Mean no. pairwise differences	Nucleotide diversity (*π*)	Tajima's *D*	Fu's *F_S_*	Mean no. pairwise differences	Nucleotide diversity (*π*)	Tajima's *D*	Fu's *F_S_*	Mean no. pairwise differences	Nucleotide diversity (*π*)	Tajima's *D*	Fu's *F_S_*
**SE Australia Shallow**	0.50 (0.52)	0.002 (0.002)	−0.612	0.172	nd	nd	nd	nd	0.67 (0.63)	0.001 (0.001)	1.633	0.540
**SE Australia Mid**	0	0	0		1.71 (1.11)	0.007 (0.005)	−0.503	−0.155	nd	nd	nd	nd
**SE Australia Deep**	1.29 (0.82)	0.004 (0.003)	−0.462	−1.119	1.35 (0.85)	0.005 (0.004)	−1.214	−6.628***	0.8 (0.68)	0.001 (0.001)	−0.973	−0.829
**New Zealand Shallow**	0	0	0	-	1.03 (0.71)	0.004 (0.003)	−1.046	−4.255***	1.04 (0.72)	0.002 (0.001)	0.794	0.027
**New Zealand Mid**	0	0	0	-	0.3 (0.3)	0.001 (0.001)	−0.941	−1.004	1.27 (0.85)	0.002 (0.002)	−1.164	−2.223*
**Chile Shallow**	0.38 (0.38)	0.001 (0.001)	0	−0.918	1.63 (1.01)	0.005 (0.004)	−1.034	−2.068	0.52 (0.46)	0.001 (0.001)	1.505	1.405
**Overall**	1.08 (0.72)	0.004 (0.003)	-	-	1.31 (0.83)	0.005 (0.003)	-	-	3.39 (1.76)	0.006 (0.003)	-	-

Numbers in parentheses are standard deviations. nd = no data.

Although mitochondrial DNA is often considered to evolve very slowly in corals, and not to be informative for intra-specific comparisons [24], [25], [26] the mitochondrial control region was the most variable of the three DNA regions studied here. The final MtC data set included 258 bp of sequence from 108 individuals, and contained 26 haplotypes. There were 24 variable sites (9.3% variation), with an average of 1.31±0.83 pairwise differences between sequences. Nucleotide diversity was still low (0.005±0.003) although higher than for 16S sequence.

Overall, the ITS sequence was moderately variable. The final ITS data set included 581 bp of sequence from 60 individuals. There were 13 variable sites (+2 indels) representing 2.5% variation, although we only found 14 unique haplotypes across SE Australia, New Zealand and Chile with an average of 3.39±1.76 pairwise differences between sequences. Across the three components of the ITS, the ITS2 was the most variable section. Of the 193 bp of ITS2 sequenced there were 9 variable sites (4.7% variation), reinforcing the usefulness of this DNA region for population-level analysis in corals. Of the 225 bp of ITS1 sequence there were only 6 variable sites (including 2 indels) representing 2.7% variation. The 163 bp of 5.8S was invariant across all samples. Nucleotide diversity (0.006±0.003) was higher in ITS than the other two DNA regions.

Estimates of Tajima's *D* and Fu's *F_s_* were mixed, with a range of positive and negative values (Table 1). However no values of Tajima's *D* varied significantly from values expected under selective neutrality, and only 3 of the 14 values of Fu's *F_s_* were statistically significant. Taken together, this shows no evidence of selection or recent change in population size for *D. dianthus* in any of the study areas.

### Association between genotype and geographic region

**Figure 2:**
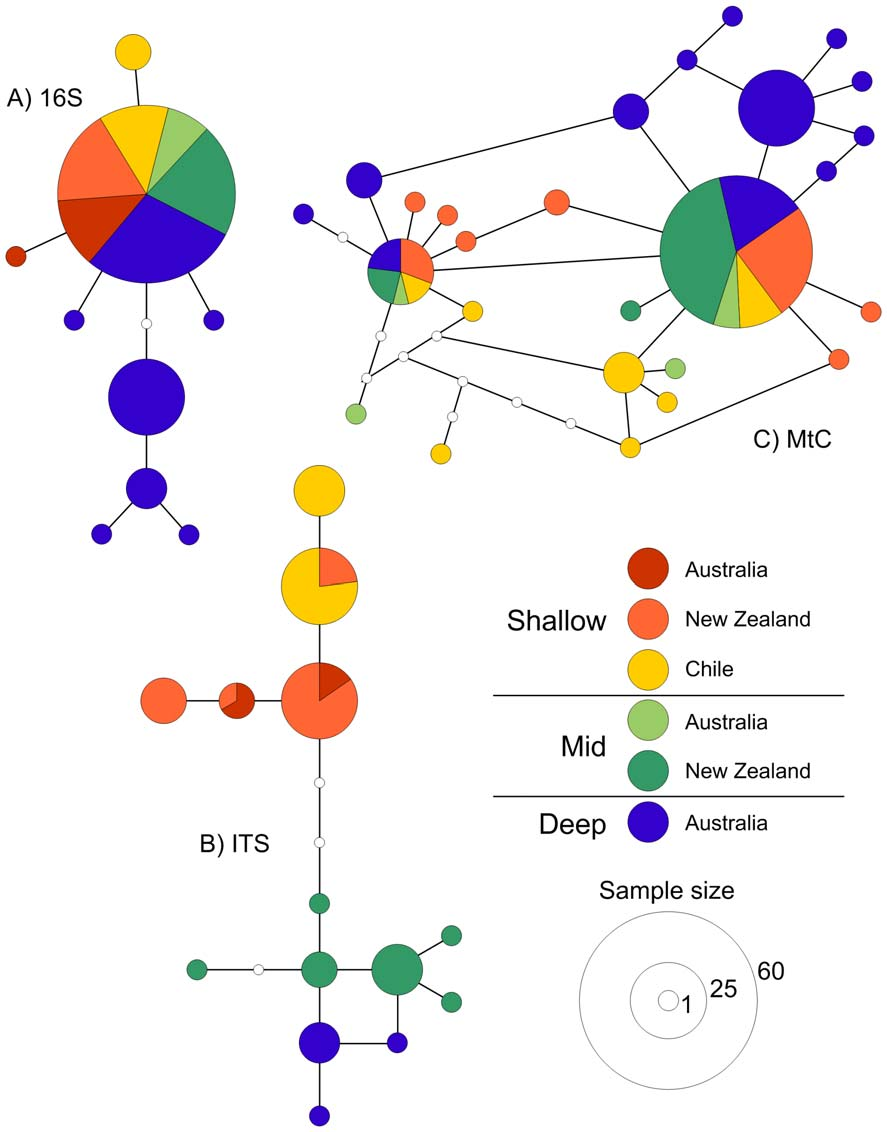
Haplotype networks based on three DNA regions for *Desmophyllum dianthus* from SE Australia, New Zealand and Chile.

Overall there were no trends in nucleotide diversity patterns across geographic regions, with similar levels of diversity recorded from SE Australia, New Zealand and Chile for each of the different gene regions (Table 1). Equally, for 16S and MtC, parsimony networks showed the data set was dominated by one or two common, presumably ancestral, haplotypes that occurred in all three geographic regions (Figs 2a & 2c). Similarly, there were multiple ITS haplotypes that occurred in at least two of the geographic regions (Fig. 2b).

Interestingly, haplotype networks showed a strong link between genotype and depth for all three DNA regions. The ITS network shows a clear distinction between shallow (<600 m), mid (1000–1500 m) and deep water (>1500 m) populations of *D. dianthus* (Fig. 2b) with no haplotypes found in more than one depth stratum, suggesting genetic differentiation is more tightly linked to depth than geographic region. For 16S and MtC networks, the deep corals are quite distinct from the mid and shallow corals and most derived haplotypes are unique to a depth within a geographic region. For example, 26 of the 28 MtC haplotypes are only found at a single depth and of these five are represented by more than one individual within that depth stratum (Fig. 2c). Similarly, in the 16S network, eight of the nine haplotypes were found only in one depth stratum with three of these represented by n>4. Despite a lack of replication across all depths and regions for all three gene regions, there is a consistent pattern suggesting that *D. dianthus* populations across the Southern Ocean have a common ancestor but that populations at different depths within the geographic regions are isolated and have begun to diverge from each other.

The physical separation of sampling sites of *D. dianthus* within each region was tens to hundreds of kilometres (Fig. 1). However we found no differences among *D. dianthus* populations from different sites at similar depths within regions, with most individuals from a region/depth sharing one or two common haplotypes (Fig. 2). Limited replication prohibited statistical tests of within-region/depth variation, but studies of other deep-sea coral taxa indicate that DNA sequence data may be less informative at these smaller spatial scales [8]. Subsequently, we pooled data into depth categories within each region for larger-scale statistical comparisons.

### Genetic differentiation and gene flow among regions and depths

Comparisons among geographically widespread populations of *Desmophyllum dianthus* showed high and statistically significant levels of genetic differentiation consistent with limited gene flow and isolation. Geographically separated populations from SE Australia, New Zealand and Chile were genetically subdivided based on sequence data from all three DNA regions (16S *F_ST_* = 0.31, p<0.001; MtC *F_ST_* = 0.19, p<0.001 and ITS *F_ST_* = 0.36, p<0.001).

Depth was a major component of the genetic differentiation with the strongest pattern of depth differentiation from the ITS sequence data (Table 2). Due to limited replication across all spatial scales and for all gene regions we were unable to do a full hierarchical analysis, however we computed pairwise *F_ST_* values among all sites and depths and found as much (and often more) genetic differentiation between populations from different depth strata within a region as we did among populations at the same depth but separated by thousands of kilometres of ocean. For example, between shallow-water SE Australian and New Zealand populations of *D. dianthus* genetic differentiation is low (i.e. *F_ST_* = 0.06 p>0.05 based on ITS) despite the sampling locations being on opposite sides of the Tasman Sea. In contrast, within SE Australia, shallow water *D. dianthus* populations are very distinct from those in deep water (*F_ST_* = 0.315 p<0.05 based on ITS), and this is the case even where shallow and deep individuals were sampled on the same seamount or on nearby seamounts (i.e. separated by hundreds of metres to <10 kms). The same pattern is apparent between shallow and mid-water populations in New Zealand (e.g. *F_ST_* = 0.35 p<0.001 based on ITS) that are separated by <200 km horizontal distance.

**Table 2:** Pairwise *F_ST_* values among populations of *Desmophyllum dianthus* from SE Australia, New Zealand and Chile collected from shallow (<600 m), mid (1000–1500 m) and deep (>1500 m) depth strata.

	SE Australia Deep	SE Australia Mid	SE Australia Shallow	New Zealand Shallow	New Zealand Mid
**16S**					
SE Australia Mid	**0.348**				
SE Australia Shallow	0.18	0.245			
New Zealand Shallow	**0.354**	0	0.271		
New Zealand Mid	**0.366**	0	0.316	0	
Chile Shallow	**0.21**	**0.26**	0.037	**0.283**	**0.306**
**MtC**					
SE Australia Mid	**0.14**				
SE Australia Shallow	x	x			
New Zealand Shallow	**0.16**	0	x		
New Zealand Mid	**0.323**	0.113	x	**0.081**	
Chile Shallow	**0.175**	0.07	x	**0.106**	**0.303**
**ITS**					
SE Australia Mid	x				
SE Australia Shallow	**0.315**	x			
New Zealand Shallow	**0.349**	x	0.063		
New Zealand Mid	**0.312**	x	**0.324**	**0.348**	
Chile Shallow	**0.419**	x	**0.435**	**0.37**	**0.402**
**N_e_m**					
SE Australia Mid	1.002				
SE Australia Shallow	0.841	0.770			
New Zealand Shallow	0.745	high	2.195		
New Zealand Mid	0.503	high	0.531	1.652	
Chile Shallow	0.822	2.016	3.416	1.056	0.505

*F_ST_* values in bold represent significant departures from values expected under panmixia (p<0.05). X denotes test not done as insufficient data for the comparison.

Considering all geographic regions and depths most populations were significantly subdivided from each other based on *F_ST_* data from at least one DNA region (Table 2), but with the exception of the mid-depth samples from SE Australia (which were significantly differentiated from only the Chilean populations), and the shallow populations from Australia and New Zealand which were genetically indistinct. However estimates of gene flow among sites calculated both by *F_ST_* and coalescence methods were low, and indicate effectively no ongoing gene flow among all the populations studied. Values of *N_e_m* ranged from 0.5 to 3.4 based on *F_ST_* (Table 2) but were <1 among all populations based on coalescent migration estimates (Table 3). We did not detect any variation in directional gene flow among populations, with source/recipient estimates not statistically significant from each other for any population pairs (Table 3). Estimates of effective population size (Θ) were similar and very low for all populations and ranged from 0.0001 to 0.0009.

**Table 3:** Estimates of bi-directional gene flow (*N_e_m*) among populations of *Desmophyllum dianthus* from SE Australia, New Zealand and Chile collected from shallow (<600 m), mid (1000–1500 m) and deep (>1500 m) depth strata as estimated using LAMARC.

ITS	Recipient population					
Source population	SE Australia Deep	SE Australia Mid	SE Australia Shallow	New Zealand Shallow	New Zealand Mid	Chile Shallow
**SE Australia Deep**	-	x	0.018	0.009	0.236	0.005
**SE Australia Mid**	x	-	x	x	x	x
**SE Australia Shallow**	0.004	x	-	0.311	0.014	0.114
**New Zealand Shallow**	0.005	x	0.486	-	0.007	0.133
**New Zealand Mid**	0.113	x	0.041	0.018	-	0.021
	0.006	x	0.2	0.13	0.004	-
**Chile Shallow**						

mtDNA	Recipient population					
Source population	SE Australia Deep	SE Australia Mid	SE Australia Shallow	New Zealand Shallow	New Zealand Mid	Chile Shallow
**SE Australia Deep**	-	0.535	x	0.073	0.028	0.187
**SE Australia Mid**	0.161	-	x	0.066	0.026	0.356
**SE Australia Shallow**	x	x	-	x	x	x
**New Zealand Shallow**	0.082	0.14	x	-	0.027	0.094
**New Zealand Mid**	0.079	0.134	x	0.068	-	0.091
	0.236	0.855	x	0.077	0.029	-
**Chile Shallow**						

Analysis for ITS was done separately from the analysis of the mtDNA (16S + MtC) to incorporate recombination. There was no statistically significant difference among any of the gene flow estimates. X denotes test not done as insufficient data for the comparison.

### Morphological variation among geographic regions

**Figure 3:**
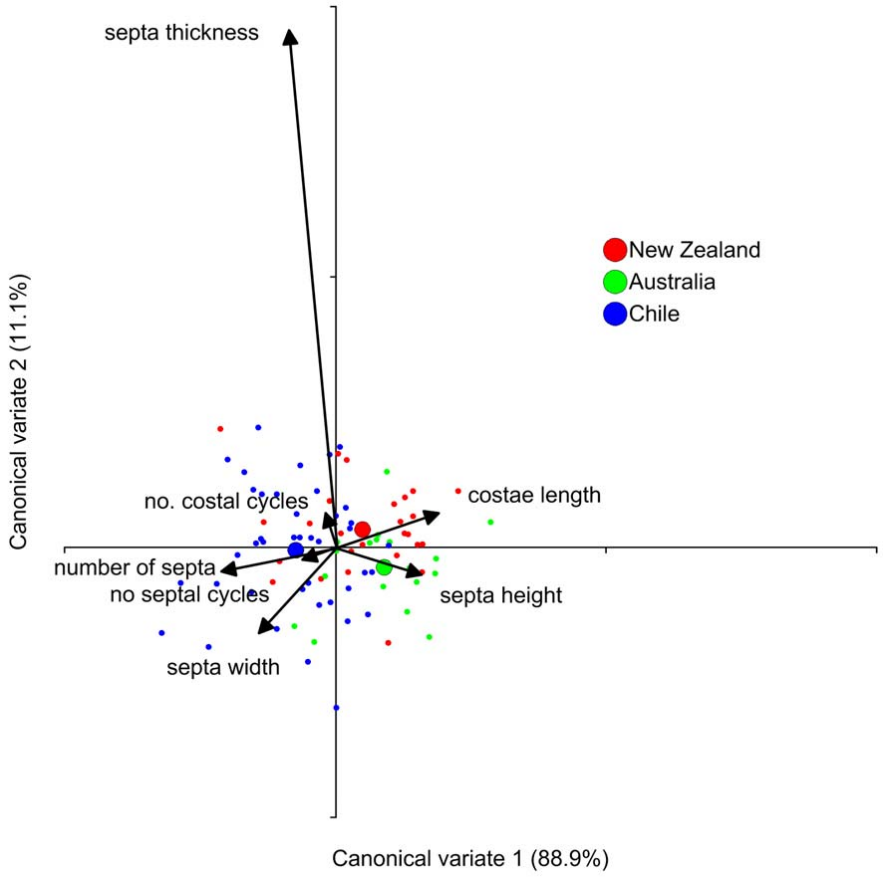
Results from Canonical Discriminant Analysis (CDA) of eight skeletal characters of *Desmophyllum dianthus* from SE Australia, New Zealand and Chile. Larger circles represent group means for each geographic region. The bi-plot is overlayed on the data graph and shows relative contributions of each of the skeletal characters to the separation of the groups. The character corallite size is not shown as part of the bi-plot as it contributed almost nothing to the separation of the groups in the analysis.

There were small, but significant, differences in the skeletal morphology of *D. dianthus* from the three geographic regions; SE Australia, New Zealand and Chile (Table 4). MANOVA based on eight skeletal characters was significant (Wilk's Lambda = 0.615, F = 2.62, df = 16, p = 0.0012) with all of the variance among groups explained with the first two canonical variables. Corals from each of the three regions, although morphologically very similar, clustered weakly as separate groups (Fig. 3) suggesting slight morphological divergence is occurring between corals in the three isolated regions. SE Australian corals tended to have taller septa than those from the other two regions, whereas New Zealand corals had longer costae. Chilean corals were overall more calcified having more costae, more septa, more septal cycles and wider and thicker septa than those from Australia or New Zealand (Fig. 3). Local environmental conditions in the shallow photic zone, including increased food supply and potential for symbiosis with endolithic algae may well facilitate skeletal deposition in fiord corals.

**Table 4:** Mean values of skeletal characters measured in *Desmophyllum dianthus* from SE Australia, New Zealand and Chile.

	n	Corallite Height	Corallite Length	Corallite Width	Total number of Septa	Septa Cycles present	Septa Height MEAN	Septa Width MEAN	Septa Thickness MEAN	Costae Cycles present	Costae Length MEAN
**New Zealand**	27	37.48	24.68	17.99	96.44	4.41	4.07	8.05	0.50	2.67	15.80
		(17.44–67.59)	(11.63–52.04)	(10.03–38.76)	(60–131)	(4–5)	(0.6875–13.896)	(4.004–17.654)	(0.244–1.418)	(2–5)	(6.54–29.166)
**SE Australia**	18	39.19	24.32	19.35	87.56	4.22	4.66	8.49	0.52	2.06	16.51
		(22.79–63.78)	(9.6–40.02)	(7.67–28.65)	(48–106)	(3–5)	(1.25–10.176)	(3.15–14.334)	(0.194–0.966)	(1–4)	(7.332–30.548)
**Chile**	41	47.23	23.19	15.26	108.22	4.46	2.54	6.62	0.36	2.73	17.49
		(11.55–116.88)	(8.85–42.21)	(7.78–22.96)	(65–178)	(4–5)	(0.67–6.154)	(3.234–10.562)	(0.15–0.762)	(1–5)	(4.484–86.116)

Ranges are in parentheses, n is the total number of individuals measured. All measurements are in mm.

## Discussion

### Isolation

Our study has provided evidence that populations of the deep-sea coral *Desmophyllum dianthus* are isolated and that there is limited contemporary gene flow between SE Australian, New Zealand and Chilean populations. However we also found evidence of strong depth stratification in *D. dianthus* populations within SE Australia and New Zealand. These results indicate that larval dispersal rarely occurs across oceanic basins, but importantly that there is no vertical exchange of larvae even across as little as several hundred of metres of depth on the same or neighbouring seamounts.

Limited dispersal in deep-sea corals is consistent with data from shallow water corals, which indicate populations are maintained via self-seeding with only limited larval dispersal from the natal site (e.g. [27], [28]). For other deep sea coral species, genetic data has also shown limited dispersal across large geographic scales [8], [29] although connectedness appears to vary among taxa because some species show genetic homogeneity across thousands of kilometres [8], [9], [30]. Likewise, genetic differentiation in other deep-sea marine invertebrates is highly variable although this may well be related to the low statistical power of many studies (e.g. [31]) linked to the cost and difficulty of replicated sampling at depth.

The apparent lack of vertical dispersal of *D. dianthus* larvae within geographic regions is surprising given the species' wide depth distribution. We were not able to compare all depths in all regions due to the limitations of deep-sea sampling, although genetic structure with depth has also been reported in other deep-sea invertebrates including ophiuroids on NW Atlantic seamounts [7], the Atlantic bivalve *Deminucula atacellana* [32], Southern Ocean ostracods [33] and isopods [34], hence we predict that this pattern is likely to be widespread. Ecological and physical conditions that occur with depth have been invoked as isolating mechanisms leading to speciation in marine invertebrates such as amphipods [35] and the dynamics of fluctuating oxygen-minimum zones may also act as barriers to gene flow in the deep-sea resulting both in genetic divergence and ultimately speciation [36]. There is no evidence of speciation within our data i.e. the low levels of morphological and genetic variation between regions and depths and the single parsimony network indicates that present day *D. dianthus* across the Southern Hemisphere should be considered a single species. However the *D. dianthus* populations we sampled in shallow, mid and deep water exist in distinct water masses corresponding with the Subantarctic Mode Water, Antarctic Intermediate Water and a mix of Circumpolar Deep and North Pacific Deep Waters respectively (e.g. [37]). These water masses are strongly depth-stratified around SE Australia and New Zealand (e.g. Subantarctic Mode Water occurs to approximately 900 m depth, the Antarctic Intermediate water from ∼900–1500 m depth with the deep water masses >1500 m depth) and our genetic results are thus consistent with oceanographic patterns indicating that planktonic larvae will be retained within the natal water mass, but rarely cross between water masses in the vertical plane. Equally, although the direction of flow of each water mass could facilitate larval dispersal among regions i.e. Intermediate Waters are contiguous and flow eastward across the Tasman Sea, and the Subantarctic Mode Water flows from SE Australia via New Zealand to Chile, distances are great and unlikely to be covered during the short life span of a coral larvae [8].

### Low levels of variation

The observed patterns of morphological and genetic differences among *D. dianthus* populations are consistent with a model of isolation and subsequent divergence. However levels of genetic and morphological variation in *D. dianthus* populations from SE Australia, New Zealand and Chile were incredibly low, despite their apparent isolation, suggesting there may be few selective pressures leading to divergence and limited localised adaptation. Indeed there was no strong signal of selection evident in the genetic data, or of rapid population expansion. Notably, the nuclear ITS region was more informative for distinguishing between geographically and bathymetrically isolated populations than either of the mtDNA regions. This finding is consistent with the recognised slow rates of mitochondrial evolution in Anthozoans relative to nuclear DNA [24], [25], [26] as well as the growing literature on the importance of ITS for coral phylogeny, phylogeography and populations genetics (e.g [8], [38], [39], [40]).

Given the slow evolutionary rate of mtDNA in corals, which are estimated to be up to 10 times less than nuclear DNA [26], [41], it may be that the presence of a few common mtDNA genotpyes across all populations – especially of the more conserved 16S – reflects historic links. Australia, New Zealand and Chile were originally part of Gondwana, and became separated early in the Cretaceous period around 130 million years ago. We do not know whether *Desmophyllum* existed during the Cretaceous, but certainly scleractinian corals are known from that period, including the closely related *Coelosmilia* sp. which may well be an ancestor of the present day *D. dianthus* [42]. Additionally recent phylogenetic studies [43] suggest the family Caryophyllidae (to which the genus *Desmophyllum* belongs) is monophyletic and thus may well have very ancient roots. Furthermore, *D. dianthus* is long lived, with individuals likely to be tens to hundreds of years old [44], [45] and hence the long generation times of corals may well slow the divergence of isolated populations. The slow rate of mtDNA evolution in combination with long generation times will likely confound the identification of evolutionary patterns and mask ecological processes in *D. dianthus* (i.e. within depths and regions) and these may only become evident in studies involving rapidly evolving nuclear markers such as microsatellites.

### Susceptibility to climate change and management implications

That deep populations are genetically distinct from shallow populations has important ramifications for conservation management under environmental change. While depth structuring in species diversity and community composition in the deep sea is accepted and relatively well studied (e.g. [46], [47], [48]) the lack of gene flow between depths has had relatively little attention to date. One exception is Costantini et al's. [49] report of genetic differences between shallow (<40 m) and deep (500–600 m) colonies of the precious red coral *Corallium rubrum*. However their limited sample sizes preclude strong conclusions about the role of depth in structuring populations of that species. Additionally, Eytan et al. [41] showed very high levels of genetic differentiation between deep (>70 m) and shallow populations in the coral genus *Oculina* – but concluded that this most likely represents recent speciation. Indeed for some species that have been considered eurybathic, molecular analysis is showing that populations at different depths most likely represent different species (e.g. [33], [34]) and this growing literature, along with our data on *D. dianthus*, emphasises that depth will be an important mechanism driving isolation in the sea.

Critically, for conservation planning, the lack of connectivity between populations at different depths will have consequences for the fate of species and populations affected by environmental change. Climate change models predict that the Aragonite Saturation Horizon will become shallower as the oceans acidify and temperatures increase, and thus deep-sea corals may face a huge habitat loss [5]. Tittensor *et al.* [6] predicted that habitat suitable for stony corals would reduce globally by up to ∼2% for seamounts and less for the surrounding seafloor. However, models predicted that certain areas would be particularly affected, mainly the North Atlantic (6–14% reduction in habitat suitability), as well as areas around New Zealand and Australia in the Southern Hemisphere. These authors suggested that the shallower waters on the summits and upper flanks of seamounts could provide a potential refuge for stony corals from the negative effects of ocean acidification. Their hypothesis is based upon the assumption that corals are easily able to disperse vertically to the most proximal suitable habitat. For some coral groups, shallow water populations are considered to have arisen from deep water descendants [50] but this has occurred over evolutionary rather than ecological timescales. Certainly the results of our study challenge the notion of seamounts as biological refuges for stony corals within the timeframe expected for climate change effects in the deep sea (i.e. <100 years, [4]). Furthermore if the ASH rises to ∼200 m depth by 2099 as predicted [5], for deep-sea corals off Tasmania this will eliminate all seamount habitats because there are no known seamounts off temperate Australia, and none expected to be discovered, that extend to within 200 m of the sea surface. While those corals that currently exist in shallow water are likely to persist, the lack of vertical dispersal suggests those populations at depths >200 m are unlikely to find refuge in shallow water and may well be lost. Because much of the genetic diversity we have found in *D. dianthus* is associated with the deepest populations in SE Australia, the loss of this genetic diversity is a real risk associated with the consequences of climate change.

The lack of vertical dispersal of coral larvae will also affect the potential for recovery of deep-sea corals directly affected by anthropogenic impacts such as fishing and mining. Deep-sea areas below depths accessed by fishing or mining technologies have been considered as potential refuges for deep-sea species and a source of recruits to aid in the recovery of exploited populations [51], [52]. Similarly, it has been proposed that deep areas of the ocean could be closed to human activities for the purpose of providing propagules to help maintain vulnerable populations at shallower depths [53]. However, if vertical dispersal is limited, then deep populations may not provide the anticipated source of propagules for recolonisation of impacted areas. A lack of recruits from deep refuges, even if closely adjacent, would be an additional factor in the slow recovery of deep water coral populations at shallower locations, such as on seamounts, that have previously been heavily impacted [54], [55]. In this study we were only able to compare *D. dianthus* from a few depths and across just part of its known range; it is therefore important that future studies are undertaken to explore the extent of depth-stratification within this and other deep water coral species. Until more is known about how and why cold-water coral populations may be restricted to specific depth zones, the protection of intact habitats over a range of depths, especially <1500 m depth and ideally <200 m, remains a conservation management priority in the deep ocean.

## Supporting Information

Table S1 Summary of the of *Desmophyllum dianthus* specimens from SE Australia, New Zealand and Chile that were sequenced in this study.
(DOCX)

## References

[1] Hoegh-Guldberg O, Bruno JF (2010). The Impact of Climate Change on the World's Marine
Ecosystems.. Science.

[2] Vezina A, Hoegh-Guldberg O (2008). Introduction: Effects of ocean acidification on marine
ecosystems.. Mar Ecol Prog Ser.

[3] Orr JC, Fabry VJ, Aumont O, Bopp L, Doney SC (2005). Anthropogenic ocean acidification over the twenty-first century
and its impact on calcifying organisms.. Nature.

[4] Guinotte JM, Fabry VJ (2008). Ocean acidification and its potential effects on marine
ecosystems..

[5] Guinotte JM, Orr J, Cairns S, Freiwald A, Morgan L (2006). Will human-induced changes in seawater chemistry alter the
distribution of deep-sea scleractinian corals?. Front Ecol Environ.

[6] Tittensor DP, Baco AR, Hall-Spencer JM, Orr JC, Rogers AD (2010). Seamounts as refugia from ocean acidification for cold-water
stony corals.. Mar Ecol.

[7] Cho W, Shank TM (2010). Incongruent patterns of genetic connectivity among four ophiuroid
species with differing coral host specificity on North Atlantic
seamounts.. Mar Ecol.

[8] Miller K, Williams A, Rowden AA, Knowles C, Dunshea G (2010). Conflicting estimates of connectivity among deep-sea coral
populations.. Mar Ecol.

[9] Baco AR, Shank TM, Freiwald A, Roberts JM (2005). Population genetic structure of the Hawaiian precious coral
Corallium lauuense (Octocorallia : Coralliidae) using
microsatellites.. Cold-Water Corals and Ecosystems.

[10] Cairns SD (1994). Scleractinia of the temperate North Pacific.. Smithsonian Contributions to Zoology.

[11] Försterra G, Häussermann V (2003). First report on large scleractinian (Cnidaria: Anthozoa)
accumulations in cold-temperate shallow water of south Chilean
fjords.. Zoologische Mededelingen Leiden.

[12] Grange KR, Singleton RJ, Richardson JR, Hill PJ, Main WD (1981). Shallow rock wall biological associations of some southern fiords
of New Zealand.. New Zeal J Zool.

[13] Häussermann V, Forsterra G, George RY, Cairns SD (2007). Large assemblages of cold-water corals in Chile: a summary of
recent findings and potential impacts.. Conservation and adaptive management of seamount and deep-sea coral
ecosystems.

[14] Le Goff-Vitry MC, Rogers AD, Baglow D (2004). A deep-sea slant on the molecular phylogeny of the
Scleractinia.. Mol Phylogenet Evol.

[15] White TJ, Bruns T, Lee S, Taylor J, Innis M, Gelfand D, Swinsky J, White TJ (1990). Amplification and direct sequencing of fungal ribosomal RNA genes
for phylogenetics.. PCR protocols: a guide to methods and applications.

[16] Kumar S, Tamura K, Nei M (2004). MEGA3: Integrated software for Molecular Evolutionary Genetics
Analysis and sequence alignment.. Brief Bioinform.

[17] Excoffier L, Laval G, Schneider S (2005). Arlequin ver 3.0: An integrated software package for population
genetics data analysis.. Evol Bioinform.

[18] Clement M, Posada D, Crandall KA (2000). TCS: a computer program to estimate gene
geneologies.. Mol Ecol.

[19] Wright S (1931). Evolution in Mendelian populations.. Genetics.

[20] Kuhner MK (2006). LAMARC 2.0: maximum liklihood and Bayesian estimation of
population parameters.. Bioinformatics.

[21] Posada D (2008). jModelTest: Phylogenetic Model Averaging.. Mol Biol Evol.

[22] Swofford DL (1998). Phylogenetic analysis using Parsimony (*and other methods). 4
ed.

[23] Cairns SD (1995). The Marine Fauna of New Zealand: Scleractinia (Cnidaria:
Anthozoa)..

[24] Shearer TL, Van Oppen MJH, Romano SL, Worheide G (2002). Slow mitochondrial DNA sequence evolution in the Anthozoa
(Cnidaria).. Mol Ecol.

[25] Hellberg ME (2006). No variation and low synonymous substitution rates in coral mtDNA
despite high nuclear variation.. BMC Evol Biol.

[26] Chen IP, Tang CY, Chiou CY, Hsu JH, Wei NV (2009). Comparative Analyses of Coding and Noncoding DNA Regions Indicate
that Acropora (Anthozoa: Scleractina) Possesses a Similar Evolutionary Tempo
of Nuclear vs. Mitochondrial Genomes as in Plants.. Mar Biotechnol.

[27] Ayre DJ, Hughes TP (2000). Genotypic diversity and gene flow in brooding and spawning corals
along the Great Barrier Reef, Australia.. Evolution.

[28] Miller KJ, Ayre DJ (2008). Protection of Genetic Diversity and Maintenance of Connectivity
among Reef Corals within Marine Protected Areas.. Cons Biol.

[29] Le Goff-Vitry MC, Pybus OG, Rogers AD (2004). Genetic structure of the deep-sea coral *Lophelia
pertusa* in the northeast Atlantic revealed by microsatellites
and internal transcribed spacer sequences.. Mol Ecol.

[30] Smith PJ, McVeagh SM, Mingoia JT, France SC (2004). Mitochondrial DNA sequence variation in deep-sea bamboo coral
(Keratoisidinae) species in the southwest and northwest Pacific
Ocean.. Mar Biol.

[31] Audzijonyte A, Vrijenhoek RC (2010). When gaps really are gaps: Statistical phylogeography of
hydrothermal vent invertebrates.. Evolution.

[32] Zardus JD, Etter RJ, Chase MR, Rex MA, Boyle EE (2006). Bathymetric and geographic population structure in the
pan-Atlantic deep-sea bivalve Deminucula atacellana (Schenck,
1939).. Mol Ecol.

[33] Brandao SN, Sauer J, Schon I (2010). Circumantarctic distribution in Southern Ocean benthos? A genetic
test using the genus Macroscapha (Crustacea, Ostracoda) as a
model.. Mol Phylogenet Evol.

[34] Raupach MJ, Malyutina M, Brandt A, Wagele JW (2007). Molecular data reveal a highly diverse species flock within the
munnopsoid deep-sea isopod Betamorpha fusiformis (Barnard, 1920) (Crustacea
: Isopoda : Asellota) in the Southern Ocean.. Deep-Sea Res Pt II.

[35] France SC, Kocher TD (1996). DNA sequencing of formalin-fixed crustaceans from archival
research collections.. Mol Mar Biol Biotech.

[36] Rogers AD (2000). The role of the oceanic oxygen minima in generating biodiversity
in the deep sea.. Deep-Sea Res Pt II.

[37] Schmitz WJ (1996). On the World Ocean Circulation: Volume 1..

[38] Chen CA, Chang CC, Wei NV, Chen CH, Lein YT (2004). Secondary structure and phylogenetic utility of the ribosomal
internal transcribed spacer 2 (ITS2) in scleractinian
corals.. Zool Stud.

[39] Coleman AW, van Oppen MJH (2008). Secondary Structure of the rRNA ITS2 Region Reveals Key
Evolutionary Patterns in Acroporid Corals.. J Mol Evol.

[40] Duenas LF, Sanchez JA (2009). Character lability in deep-sea bamboo corals (Octocorallia,
Isididae, Keratoisidinae).. Mar Ecol Prog Ser.

[41] Eytan RI, Hayes M, Arbour-Reily P, Miller M, Hellberg ME (2009). Nuclear sequences reveal mid-range isolation of an imperilled
deep-water coral population.. Mol Ecol.

[42] Stolarski J, Meibom A, Przenioslo R, Mazur M (2007). A Cretaceous scleractinian coral with a calcitic
skeleton.. Science.

[43] Kitahara MV, Cairns SD, Stolarski J, Blair D, Miller DJ (2010). A Comprehensive Phylogenetic Analysis of the Scleractinia
(Cnidaria, Anthozoa) Based on Mitochondrial CO1 Sequence
Data.. PLoS ONE.

[44] Adkins JF, Henderson GM, Wang SL, O'Shea S, Mokadem F (2004). Growth rates of the deep-sea scleractinia Desmophyllum
cristagalli and Enallopsammia rostrata.. Earth Planet Sc Lett.

[45] McCulloch M, Montagna P, Forsterra G, Mortimer G, Haussermann V (2005). Uranium-series dating and growth rates of the cool-water coral
Desmophyllum dianthus from the Chilean fjords..

[46] Carney RS (2005). Zonation of deep biota on continental margins..

[47] Arantes RCM, Castro CB, Pires DO, Seoane JCS (2009). Depth and water mass zonation and species associations of
cold-water octocoral and stony coral communities in the southwestern
Atlantic.. Mar Ecol Prog Ser.

[48] Williams A, Bax NJ, Kloser RJ, Althaus F, Barker B (2009). Australia's deep-water reserve network: implications of
false homogeneity for classifying abiotic surrogates of
biodiversity.. ICES J Mar Sci.

[49] Costantini F, Taviani M, Remia A, Pintus E, Schembri PJ (2010). Deep-water Corallium rubrum (L., 1758) from the Mediterranean
Sea: preliminary genetic characterisation.. Mar Ecol.

[50] Lindner A, Cairns SD, Cunningham CW (2008). From Offshore to Onshore: Multiple Origins of Shallow-Water
Corals from Deep-Sea Ancestors.. PLoS ONE.

[51] Morato T, Watson R, Pitcher TJ, Pauly D (2006). Fishing down the deep.. Fish Fish.

[52] Williams A, Schlacher TA, Rowden AA, Althaus F, Clark MR (2010). Seamount megabenthic assemblages fail to recover from trawling
impacts.. Mar Ecol.

[53] Davies AJ, Roberts JM, Hall-Spencer J (2007). Preserving deep-sea natural heritage: Emerging issues in offshore
conservation and management.. Biol Conserv.

[54] Clark MR, Koslow JA, Pitcher TJ, Morato T, Hart PJB, Clark MR, Haggan N (2007). Impacts of fisheries on seamounts;.

[55] Althaus F, Williams A, Schlacher TA, Kloser RJ, Green MA (2009). Impacts of bottom trawling on deep-coral ecosystems of seamounts
are long-lasting.. Mar Ecol Prog Ser.

