# Supplementary material for: Out of Their Depth? Isolated Deep Populations of the Cosmopolitan Coral Desmophyllum dianthus May Be Highly Vulnerable to Environmental Change
Source: PLoS One. 2011 May 18;6(5):e19004. doi: 10.1371/journal.pone.0019004 (PMC3097177; doi:10.1371/journal.pone.0019004)
Supplement: Table S1 — Summary of the of Desmophyllum dianthus specimens from SE Australia, New Zealand and Chile that were sequenced in this study. (DOCX) [file pone.0019004.s001.docx]

Table S1. Summary of the of *Desmophyllum dianthus* specimens from SE Australia, New Zealand and Chile that were sequenced.

|  | **Location** | **Latitude** | **Longitude** | **Depth^1^ (m)** | **Collection date** | **16S** | **ITS** | **MtC** |
| --- | --- | --- | --- | --- | --- | --- | --- | --- |
| **SE Australia** | Cascade Plateau | -43.92231 | 150.46517 | 590-660 | 10/04/2007 | 4 | 4 | 0 |
|  | Cascade Plateau | -43.80679647 | 150.3379197 | 2170 | 3/01/2009 | 1 | 0 | 1 |
|  | Cascade Plateau | -43.8058877 | 150.3221563 | 2279 | 3/01/2009 | 6 | 0 | 8 |
|  | Cascade Plateau | -43.80316587 | 150.320886 | 2395 | 3/01/2009 | 6 | 2 | 7 |
|  | NSW | -34.3666 | 151.3833 | 439 | 14/12/1978 | 4 | 0 | 2 |
|  | NSW | -36.9366 | 150.3833 | 1600 | 12/12/1986 | 1 | 0 | 0 |
|  | Tasmania | -44.32567 | 147.17516 | 1100-1160 | 2/04/2007 | 0 | 0 | 2 |
|  | Tasmania | -44.32245 | 147.18085 | 1100-1200 | 1/04/2007 | 1 | 0 | 0 |
|  | Tasmania | -44.32626 | 147.11893 | 1100-1200 | 3/04/2007 | 2 | 0 | 0 |
|  | Tasmania | -44.23179 | 147.47266 | 1100-1350 | 3/04/2007 | 2 | 0 | 0 |
|  | Tasmania | -44.24493 | 146.16426 | 1120-1350 | 5/04/2007 | 0 | 0 | 1 |
|  | Tasmania | -44.24496 | 146.16445 | 1120-1360 | 5/04/2007 | 0 | 0 | 1 |
|  | Tasmania | -41.2431 | 148.8258 | 1170-1380 | 2008 | 0 | 0 | 2 |
|  | Tasmania | -44.3882638 | 147.255778 | 1616 | 26/12/2008 | 1 | 0 | 2 |
|  | Tasmania | -44.38206906 | 147.2630531 | 1898 | 25/12/2008 | 4 | 1 | 4 |
|  | Tasmania | -44.38970895 | 147.27172 | 2040 | 25/12/2008 | 4 | 1 | 5 |
|  | Tasmania | -44.3005 | 147.45 | 2194 | 14/01/2009 | 12 | 0 | 0 |
|  | Tasmania | -45.36629388 | 144.6245941 | 2386 | 9/01/2009 | 13 | 1 | 17 |
|  |  |  |  |  | **Totals^2^** | **61** | **9** | **52** |
| **New Zealand** | Auckland Islands | -50.6833 | 167.679993 | 265 | 18/09/1978 | 1 | 4 | 2 |
|  | Auckland Islands | -50.678299 | 167.684998 | 300 | 18/09/1978 | 0 | 3 | 4 |
|  | Auckland Islands | -50.633301 | 167.633301 | 320-380 | 7/12/1973 | 0 | 1 | 1 |
|  | Auckland Islands | -50.696701 | 167.676697 | 339-417 | 17/09/1978 | 0 | 0 | 1 |
|  | Chatham Rise | -42.786167 | 180.014667 | 900-993 | 16/04/2001 | 0 | 0 | 1 |
|  | Chatham Rise | -42.727 | 180.1017 | 990 | 7/06/2006 | 1 | 1 | 1 |
|  | Chatham Rise | -42.7275 | 180.101 | 1000 | 7/06/2006 | 1 | 3 | 3 |
|  | Chatham Rise | -42.76534 | 180.0718 | 1005 | 2006 |  | 1 | 1 |
|  | Kermadec Ridge | -36.82833333 | 177.448 | 1118-1400 | 8/11/2004 | 2 | 1 | 2 |
|  | Macquarie Ridge | -52.4873333 | 160.4150000 | 350-560 | 9/04/2008 | 1 | 2 | 1 |
|  | Macquarie Ridge | -51.0610000 | 161.9780000 | 385-498 | 4/04/2008 | 3 | 2 | 6 |
|  | Macquarie Ridge | -51.0470000 | 162.0188333 | 398-489 | 5/04/2008 | 5 | 6 | 7 |
|  | Macquarie Ridge | -52.3975000 | 160.6566667 | 451-438 | 9/04/2008 | 2 | 2 | 2 |
|  | Macquarie Ridge | -55.3616667 | 158.4278333 | 501-630 | 15/04/2008 | 0 | 0 | 1 |
|  | Macquarie Ridge | -53.7151667 | 159.1305000 | 770-810 | 12/04/2008 | 1 | 0 | 1 |
|  | Macquarie Ridge | -53.7380000 | 159.1141667 | 925-1014 | 11/04/2008 | 1 | 2 | 2 |
|  | Macquarie Ridge | -53.7045000 | 159.1145000 | 998-1100 | 13/04/2008 | 0 | 0 | 1 |
|  | Macquarie Ridge | -50.0971667 | 163.4741667 | 1070-1123 | 1/04/2008 | 4 | 5 | 5 |
|  | Macquarie Ridge | -50.0905000 | 163.4821667 | 1077-1408 | 1/04/2008 | 0 | 0 | 5 |
|  | Macquarie Ridge | -53.7313333 | 159.1663333 | 1150-1270 | 12/04/2008 | 1 | 1 | 1 |
|  | Macquarie Ridge | -59.0241667 | 158.8666667 | 1260-1351 | 19/04/2008 | 1 |  | 1 |
|  |  |  |  |  | **Totals^2^** | **24** | **34** | **49** |
| **Chile** | Comau Fjord | -42.162 | 72.5985 | 20 | 10/01/2006 | 4 | 2 | 3 |
|  | Comau Fjord | -42.3879 | 72.4609 | 20 | 12/01/2006 | 3 | 4 | 3 |
|  | Comau Fjord | -42.4095 | 72.4243 | 22 | 6/01/2006 | 0 | 1 | 0 |
|  | Comau Fjord | -42.3315 | 72.461 | 22 | 9/01/2006 | 5 | 5 | 5 |
|  | Canal Fallos | -48.8259 | 75.0518 | 25 | 14/03/2006 | 0 | 0 | 1 |
|  | Seno Waldemar, | -48.3968 | 74.7302 | 25 | 15/03/2006 | 0 | 0 | 1 |
|  | Reñihué Fjord | -42.5462 | 72.5483 | 27 | 19/07/2006 | 0 | 0 | 1 |
|  | Reñihué Fjord | -42.5597 | 72.5356 | 30 | 19/07/2006 | 0 | 0 | 1 |
|  | Punta Llonco | -42.3439 | 72.4511 | 30 | 3/01/2006 | 3 | 6 | 3 |
|  |  |  |  |  | **Totals^2^** | **15** | **18** | **18** |

^1^ Depth ranges indicate start and finish depths for trawl samples. ^2^Due to poor preservation of some samples, amplification at all three DNA regions was not possible for all specimens (see [8]). For the final data set we endeavoured to include sequences for a similar number of individuals from each of the geographic regions where possible.
